# Choroidal Changes of Long-Term Type 1 Diabetic Patients without Retinopathy

**DOI:** 10.3390/diagnostics10040235

**Published:** 2020-04-19

**Authors:** Elvira Orduna-Hospital, Lorena Perdices, Ana Sanchez-Cano, Javier Acha, Nicolás Cuenca, Isabel Pinilla

**Affiliations:** 1 Research in Retina, Aragon Institute for Health Research (IIS Aragon), 50009 Zaragoza, Spain; elvisabi14@hotmail.com (E.O.-H.); lperdices@gmail.com (L.P.); anaisa@unizar.es (A.S.-C.); j.acha.perez@gmail.com (J.A.); 2 Department of Ophthalmology, Miguel Servet University Hospital, 50009 Zaragoza, Spain; 3 Department of Applied Physics, University of Zaragoza, 50009 Zaragoza, Spain; 4 Department of Endocrinology, Miguel Servet University Hospital, 50009 Zaragoza, Spain; 5 Department of Physiology, Genetics and Microbiology, University of Alicante, 03690 Alicante, Spain; cuenca@ua.es; 6 Department of Ophthalmology, Lozano Blesa University Hospital, 50009 Zaragoza, Spain

**Keywords:** diabetic retinopathy, choroidal thickness, choroidal volume, spectral domain optical coherence tomography (SD-OCT), swept source optical coherence tomography (SS-OCT), type 1 diabetes mellitus, long evolution

## Abstract

The aim of the study is to assess choroidal thickness (CT) and choroidal volume (CV) in 90 type 1 diabetes mellitus (DM1) patients with no diabetic retinopathy (DR) and 60 control eyes using spectral domain optical coherence tomography (SD-OCT) and swept source (SS)-OCT in the areas of the Early Treatment Diabetic Retinopathy Study (ETDRS). Mean ages were 42.93 ± 13.62 and 41.52 ± 13.05 years in the diabetic and control groups, respectively. Significant differences were obtained between both groups with Spectralis SD-OCT in all ETDRS areas and in the total CV, excluding the temporal perifoveal one. With Triton SS-OCT, statistically significant differences were obtained in the subfoveal CT and in the vertical areas. CT showed the same tendency with both OCTs, with greater CT and CV in the DM1 group than the mean values of the control group. To assess the influence of DM1 evolution in the CT modifications, DM1 patients were divided into Group 1, with less than 24 years of diagnosis, and Group 2, with ≥24 years of DM1 evolution. Using both OCTs, seven of the nine ETDRS areas and the CV had lower values in Group 2. CT and CV measured by OCT were higher in DM1 without DR. There is a choroidal thinning related to disease evolution in DM1. In patients with DM evolution greater than 24 years, the CT is statistically lower than in patients with less evolution of the disease.

## 1. Introduction

One of the main causes of vision loss worldwide is diabetic retinopathy (DR) [1]. Retinal function requires a healthy choroid to nourish the different retinal layers, providing oxygen and nutrients and thermoregulating [2]. Traditionally, the choroid could only be evaluated by ultrasonography, indocyanine green angiography and laser flowmetry, showing the blood flow and abnormalities in the choroidal vessels. No device managed to cross the retinal pigment epithelium (RPE) and show the three-dimensional anatomy of the different choroid layers. Recently, a non-invasive imaging technique, optical coherence tomography (OCT), can acquire multiple consecutive high-resolution images of retina sections, showing the different retinal layers and the optic nerve. To improve the choroidal visualization, enhanced depth imaging (EDI) with spectral-domain (SD)-OCT diminishes the vitreous details, achieving a better choroidal view by inverting the retinal image and reaching the sensitivity to distinguish the limit of the choroid with the sclera [3,4]. Other devices using a longer wavelength (1050 nm), such as the swept-source (SS)-OCT, manage to overcome the high reflectivity of the RPE and choroidal vascularization, optimizing tissue penetration and obtaining an even greater resolution and exploration speed [5].

The choroid, in addition to being essential for maintaining normal eye physiology, has been found to be involved in the etiopathogenesis of several ocular diseases, including age-related macular degeneration (AMD) and choroidal thickness (CT) diseases, polypoidal choroidal vasculopathy, central serous choroidopathy, myopia magna, and infiltrative or inflammatory diseases such as Harada’s disease [6,7,8]. The CT can be increased or decreased depending on the pathology. Several papers have described a decrease in CT in glaucomatous patients and patients with other retinal diseases, and this diminution progresses with time or with treatment [9].

There are clinical, histopathological and experimental studies that suggest a relationship between DR and choroidal involvement. Choroidal abnormalities have been described in diabetic patients, including the presence of microaneurysms, dilatations, closures or capillary modification at the choriocapillaris level with increased vascular tortuosity, capillary loss, areas of non-perfusion and even choroidal neovascularization [10,11,12,13]. In diabetic patients suffering from DR, studies have shown a decrease in CT in subjects with proliferative DR or diabetic macular edema (DME) and few changes in patients with non-proliferative DR or without DR. Breakage of the blood-retinal barrier, alteration in the vascular integrity of the retina or hemodynamic anomalies cause changes in CT in murine diabetes mellitus (DM) models [14,15]. We wanted to assess the existence of choroidal changes in type 1 DM (DM1) prior to the development of DR.

The aim of this study was to assess CT with two different OCT devices in the areas of the Early Treatment Diabetic Retinopathy Study (ETDRS) in DM1 patients without retinopathy compared with healthy subjects.

## 2. Methods

We undertook a prospective study during 2017 including 90 eyes from 90 DM1 patients without DR. The experimental protocol was approved by the local Ethics Committee for Clinical Research of Aragon (CEICA) and the evaluation was conducted in accordance with the principles of the Helsinki Declaration. Detailed consent forms were obtained from each patient.

DM1 patients were controlled by the endocrinology unit. Blood samples were analyzed every six months. Glycosylated hemoglobin (HbA1c), lipid values and arterial blood pressure were maintained under extreme control. The DM1 group was equally divided depending on the duration of the disease: from 9 to 24 years (Group 1, *n* = 46) and from 24 to 40 years (Group 2, *n* = 44). Control group was divided in two to evaluate if differences in the diabetic group were related to age.

The inclusion criteria for the DM1 group was a DM1 diagnosis and no retinal changes identified by biomicroscopy and OCT by at least two retinal specialists. The control group included age-matched healthy subjects. All subjects had a best corrected visual acuity (BCVA) over 20/25 on the Snellen chart, with refractive errors between +5.00 to −5.00 diopters, normal anterior pole examination with slit-lamp and no fundoscopy findings. Exclusion criteria was the presence of any sign of DR or any kind of retinopathy, glaucoma or intraocular pressure (IOP) over 21 mm Hg assessed by Goldman tonometry, optic nerve pathology, ocular inflammation or any ocular surgery or procedure including laser therapy, ocular trauma, anterior segment pathology or media opacification.

On each patient’s visit, a detailed familiar, systemic and ophthalmological medical history was performed. The axial length (AL) was measured with the optical biometry IOLMaster^®^500 from Carl Zeiss Meditec (Carl Zeiss Meditec, Oberkochen, Germany).

**Figure 1:**
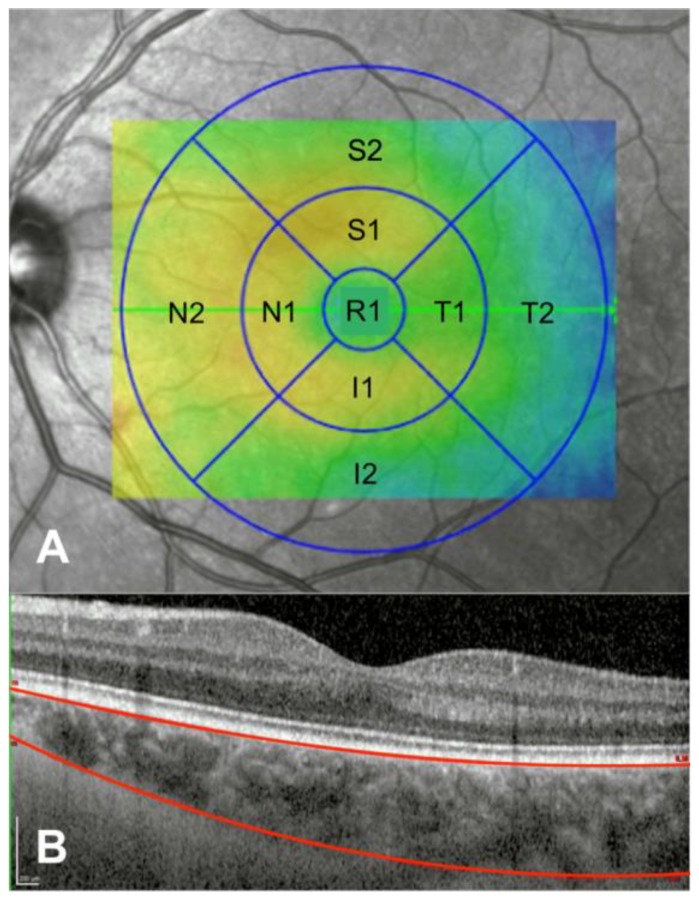
(**A**) The nine macular Early Treatment Diabetic Retinopathy Study (ETDRS) circular areas, including the fovea (1 mm, R1), temporal inner (T1), superior inner (S1), nasal inner (N1), inferior inner (I1), temporal outer (T2), superior outer (S2), nasal outer thickness (N2), and inferior outer (I2) areas, where the measurements were done. (**B**) Foveal optical coherence tomography (OCT) profile limiting the choroidal thickness with the superior line at the outer limit of the retinal pigment epithelium (RPE) layer and the inferior line at the choroid-scleral limit. Scale bar: 200 microns.

Each individual was imaged using a Spectralis SD-OCT (Heidelberg Engineering, Inc., Heidelberg, Germany) device and Deep Range Imaging (DRI) DRI-Triton SS-OCT (Topcon Corporation, Tokyo, Japan). With Spectralis SD-OCT, the volume fast macula with enhanced depth imaging (EDI) scanning protocol was performed. The subject was asked to look into the internal fixation target and Tru-Track eye tracking technology was used. Spectralis SD-OCT provides a circular macular map analysis divided in nine sectorial thickness measurements in three concentric circles with diameters of 1, 3 (inner), and 6 (outer) mm forming the 9 areas corresponding to the ETDRS [16]. The regional choroidal thicknesses (CTs) and volumes (CVs) including the fovea (1 mm, R1), the parafoveal ring with four quadrants, temporal inner (T1), superior inner (S1), nasal inner (N1), inferior inner (I1), and four perifoveal quadrants: temporal outer (T2), superior outer (S2), nasal outer thickness (N2), and inferior outer (I2) choroidal submacular thicknesses were analysed (Figure 1). The Spectralis software version was 6.8.1.0. Once the macular maps were obtained with EDI, the reference lines given by the device were manually modified, placing the line of the Internal Limiting Membrane (ILM) at the outer limit of the RPE layer, and the line marking Bruch’s Membrane (BM) was moved to the choroidscleral limit. After manual modification, the CT was shown in every ETDRS area. The quality of the scans was checked, and poor-quality scans were rejected. Images should achieve at least 25 over 40 dB. With DRI-Triton SS-OCT, a macular 6.0 × 6.0 mm three-dimensional scan was obtained, and automatic segmentation of CT was made by the device from BM to the limit between the choroid and the sclera. DRI-Triton SS-OCT includes the new SMARTTrack^TM^ tool that enhances tracking, corrects for motion, and guides the operator to reduce potential errors while acquiring the images. Only eyes with good-quality scans were included in the analysis. Good-quality DRI-Triton SS-OCT images were defined as those with a signal strength ≥70/100, and without motion artefacts, involuntary saccades, or overt misalignment of decentration. DRI-Triton SS-OCT provides the same circular macular map analysis as the Spectralis SD-OCT, which is composed of the 9 areas corresponding to the ETDRS (Figure 1).

We used two different devices because both of them are commonly used in the clinic, and they operate at different wavelengths (840 nm for SD-OCT and 1050 nm for SS-OCT) with variable penetration depth and resolution. They provide different measurements that can vary in both healthy and disease eyes. Qualitatively, DRI Triton SS-OCT shows choroidal images with higher resolution than the Spectralis SD-OCT; this fact could be due to its higher penetration depth, and subsequently, the delimitation of the rear part of the choroid seems to be more accurate. In both exams with the two different devices, the subjects were seated and properly positioned. A single and well-trained technician obtained all OCT images.

Statistical analysis was performed using the Statistical Package for the Social Sciences (SPSS 22.0, SPSS, Chicago, IL, USA). Normal distribution of the values was studied with the Kolmogorov-Smirnov test. The variables of CT and total CV were compared between two independent groups, such as the control group and the DM1 group, with the Kolmogorov-Smirnov test for two independent nonparametric samples. A *p* value <0.05 was considered statistically significant.

## 3. Results

The mean age of the 90 DM1 patients was 41.52 ± 13.05 years (22–65) and 42.41 ± 13.56 years (26–68) for the 60 healthy controls. There were no age differences (*p* = 0.361). DM1 patients were well-controlled with a mean glycosylated hemoglobin (HbA1c) of 7.76 ± 1.06%. The distribution by sex was as follows: 65% of the subjects in the control group and 46.7% in the group of DM1 patients were women. The male population was 35% in the control group and 53.3% in the diabetic group. The differences reached statistical significance (*p* = 0.019) by the greater number of women. Calculations were made to rule out that sex was a confounding variable, since no statistically significant differences were found in the CT measured by SD and SS-OCT between men and women.

Both groups had no differences in their AL (*p* = 0.908), anterior chamber depth (ACD) (*p* = 0.999) or refractive defects (*p* = 0.394). These values are shown in Table 1.

**Figure 2:**
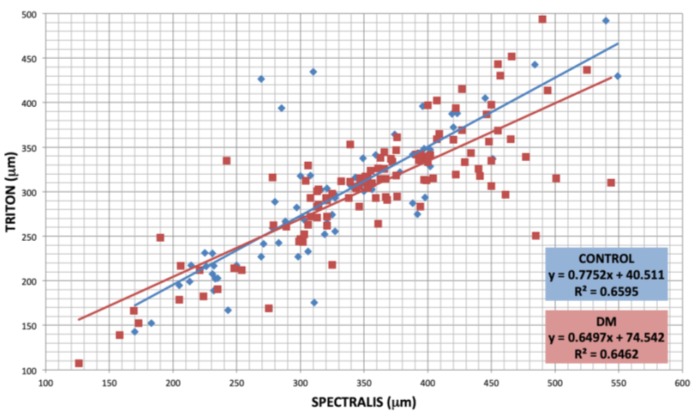
Agreement between subfoveal choroidal thickness (SFCT) measured with both devices in both groups. Control group (blue) showed a distribution following the equation for optical coherence tomography (SS-OCT) (μm) = 0.7752 × SS-OCT(μm) + 40.511, being R^2^ = 0.6595 and DM1 group (red) SS-OCT (μm) = 0.6497 × SS-OCT(μm) + 75.542, being R^2^ = 0.6462.

A linear correlation was observed in terms of subfoveal choroidal thickness (R1) between the two devices, both for the DM1 and for the control group, as shown in Figure 2.

**Figure 3:**
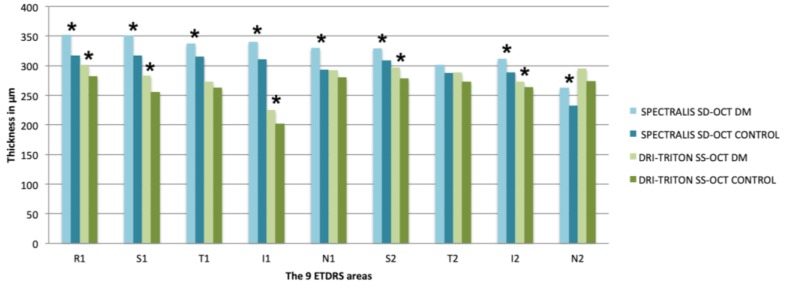
Mean of choroidal thicknesses in each ETDRS area expressed in μm measured with the Spectralis spectral domain optical coherence tomography (SD-OCT) and the deep range imaging (DRI)-Triton swept source optical coherence tomography (SS-OCT) in the type 1 diabetes Mellitus (DM1) group and the control group. Significant differences were considered as *p* < 0.05, calculated by Kolmogorov–Smirnov test for two independent nonparametric samples, are shown with *. SD-OCT, spectral domain optical coherence tomography; DRI, deep range image; SS-OCT, swept source optical coherence tomography; DM, diabetes mellitus; ETDRS, Early Treatment Diabetic Retinopathy Study; R1, central; S, superior; T, temporal; I, inferior; N, nasal. The number 1 corresponds to the 3 mm parafoveal inner circle and 2 to the 6 mm perifoveal outer circle.

In both groups and with both types of OCT equipment, the choroid was thickest in the central area (R1) and in the parafoveal areas (N1, S1, T1 and I1). CT decreased from R1 to the parafoveal and perifoveal areas, with the nasal perifoveal area (N2) being the thinnest with both devices and in both groups (Table 2 and Figure 3). The vertical areas were thicker than the horizontal areas, the upper areas were the thickest of the vertical areas and the nasal areas were the thinnest of the horizontal areas.

Significant differences were obtained between the DM1 group and the control group with Spectralis SD-OCT in all ETDRS quadrants and in the total CV (8.82 ± 2.09 mm^3^ in the DM1 group vs. 8.12 ± 1.92 mm^3^ in control group, *p* = 0.003), excluding the temporal perifoveal area (T2: 302.07 ± 71.46 μm in the DM1 group vs. 287.75 ± 60.22 μm in the control group; *p* = 0.107). CT and total CV were higher in all quadrants in the DM1 group (Table 2 and Figure 3).

Using the DRI-Triton SS-OCT, statistically significant differences were obtained in the central area (R1: 301.30 ± 64.55 μm vs. 282.32 ± 74.26 μm in diabetics vs. controls respectively; *p* = 0.025) and in the vertical areas, both perifoveal (S2: 297.05 ± 69.55 μm vs. 278.77 ± 79.90 μm, *p* = 0.040 and I2: 272.65 ± 73.86 μm vs. 263.54 ± 75.29 μm, *p* = 0.036 in diabetics vs. controls, respectively) and parafoveal (S1: 283.18 ± 68.26 μm vs. 255.81 ± 76.83 μm, *p* = 0.014 and I1: 225.23 ± 63.27 μm vs. 202.09 ± 72.93 μm, *p* = 0.016, in diabetics vs. controls, respectively). No differences were found in either the horizontal areas, or in the total CV (*p* > 0.05). CT showed the same tendency with Spectralis SD-OCT, with greater CT and CV in the DM1 group than the mean values of the control group (Table 2 and Figure 3).

Finally, to assess the influence of DM1 duration on the CT modifications, DM1 patients were divided into two groups. The mean DM1 evolution time was 24.88 ± 8.42 years. Group 1 was formed by 46 patients less than 24 years after diagnosis and Group 2 by 44 patients with ≥24 years of DM1 evolution. Control group was also divided into two, Group 1 was formed by subjects ≤38 years, and Group 2 by subjects older than 38 years.

**Figure 4:**
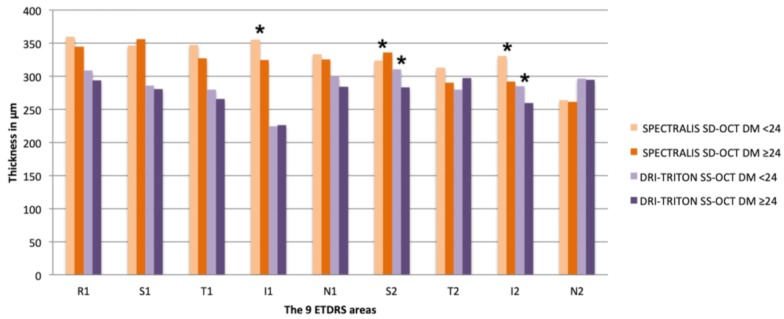
Mean of choroidal thicknesses in each ETDRS area in μm (from the BM to the choroid-scleral limit) assessed with Spectralis spectral domain optical coherence tomography (SD-OCT) and the deep range imaging (DRI)-Triton swept source optical coherence tomography (SS-OCT) in the type 1 diabetes Mellitus (DM1) group subdivided into two groups: less than 24 years and over or equal to 24 years of evolution of DM1. Significant differences were considered as *p* < 0.05, calculated by Kolmogorov–Smirnov test for two independent nonparametric samples, are shown with *. SD-OCT, spectral domain optical coherence tomography; DRI, deep range image; SS-OCT, swept source optical coherence tomography; DM, diabetes mellitus; ETDRS, Early Treatment Diabetic Retinopathy Study; R1, central; S, superior; T, temporal; I, inferior; N, nasal. The number 1 corresponds to the 3 mm parafoveal inner circle and 2 to the 6 mm perifoveal outer circle.

There were significant differences in age between both groups, with older patients having the longer evolution time (mean age Group 1, 35.65 ± 12.87 years vs. Group 2, 45.59 ± 9,96 years, *p* < 0.001). There were no differences regarding refractive status, ACD, or AL in either control or DM group (Table 3). Analyzing the results of the DM1 group according to the years of evolution of the disease with both OCT methods separately, we found significant differences in three of the vertical ETDRS quadrants, the two inferior ones and the superior perifoveal area (I1, *p* = 0.031; I2, *p* = 0.036 and S2, *p* = 0.044), using Spectralis SD-OCT. With DRI-Triton SS-OCT, differences were found only in the two vertical perifoveal areas (S2, *p* = 0.016 and I2, *p* = 0.045). Using Spectralis SD-OCT, seven out of the nine ETDRS areas and the CV had lower values in the group with longer disease evolution; similar results were shown with the DRI-Triton SS-OCT (Table 4 and Figure 4); in the control group, by using Spectralis we only found differences in T1.

## 4. Discussion

There are numerous physiological variables such as AL, refractive error, IOP, diurnal variation, other systemic vascular diseases and various drugs that could affect the CT in addition to the different age ranges of the population groups of each study. In this study, we included patients of similar age to avoid the diminution of the CT with age shown in previous works, and AL was a fundamental factor to be taken into account in the measurement of CT [17,18,19,20,21,22]. We tried to exclude confounding factors by looking for young people with good control, and considerable refractive errors were discarded to avoid bias.

Our findings show similar results as previously described in CT; CT was thickest in the subfoveal areas, becoming thinner towards the nasal or temporal areas [11,17,18,23,24].

With the Spectralis SD-OCT, significant differences were obtained between the DM1 group and the control group in all ETDRS areas and in the total CV, except in the temporal perifoveal area (T2: *p* = 0.107). We observed that the average CT and CV was higher in the DM1 group in all areas.

Statistically significant differences were obtained with the DRI-Triton SS-OCT in the central area (R1, *p* = 0.025) and in the vertical areas, both perifoveal (S2, *p* = 0.040 and I2, *p* = 0.036) and parafoveal (S1, *p* = 0.014 and I1, *p* = 0.016). However, we did not find the same differences in either the horizontal areas or in the total CV. As previously mentioned, the horizontal areas are the thinnest in both the parafoveal and perifoveal circles. It is likely that with less thickness in these areas, we would need to increase the sample size to demonstrate the same trend. We also found that the CT and CV of diabetics were higher than the average thickness and CV of the control group.

The CT obtained with Spectralis SD-OCT was higher in all ETDRS areas compared to those measured with DRI-Triton SS-OCT, which have been previously observed in a group of healthy subjects with a high correlation [25]; the same was observed in this study in both the control and the DM1 group. Factors such as thick choroids, RPE pigmentation or vascular structures can add difficulty to the establishment of the choroidal limit using SD-OCT and lead to inconsistencies between the results of the OCT systems. For eyes with these characteristics, a high-penetration OCT with a longer wavelength would be more accurate.

Tavares et al. [26] observed in their one-year follow-up study that the CT of diabetic patients without DR increased, while the thickness of the inner retinal layers and the total retina decreased, similar to the findings in our research. Several studies have analyzed CT in diabetic patients without DR, although the results have been contradictory. Esmaeelpour and Querques found that thinning of the choroid was independent of the stage of the disease, even in patients without DR [12,27,28,29]. Esmaeelpour included only 33 DM1 patients with an age similar to our study. On the other side, Querques’ patients had a mean age of 65 years. In contrast, Xu et al. found that choroidal subfoveal thickening was associated with DM in a study including 246 diabetic subjects, 23 of whom had DR. However, this difference was not related to the presence or stage of DR after adjusting for several confounding factors [30]. Vujosevic et al. did not find significant differences in CT between 102 diabetic patients and controls [13].

In the study by Tavares et al. looking at choroidal thickening at one-year follow-up, they discuss that the increase in CT observed in DM patients without DR could correspond to the presence of a choroidal vasculopathy, so DME or vascular dilatation with greater rigidity of the blood vessels may be responsible for the increased CT. We found similar results [26]. The autoregulation of the choroid is controversial [31]. In diabetic patients, Nagaoka et al. [32] showed that the choroidal blood flow could be decreased, even before any DR signs.

After development of DR, there is a tendency towards decreased CT that could correspond to vascular modifications and microvascular loss. Esmaeelpour [12] and Abadia [33] found that subfoveal CT was thinner in DM2 patients without DR and with non-proliferative DR compared to healthy controls. Kim et al. [11] evaluated the subfoveal CT at a distance of 1500 μm superior, inferior, nasal and temporal to the fovea. Unlike the previously mentioned studies [11,28,33], they found that the subfoveal CT in the presence of proliferative DR was thicker than in eyes without DR, or with mild-to-moderate and severe non-proliferative DR. However, in comparison with healthy controls, the subfoveal, temporal, nasal, superior and inferior thicknesses of the choroid decreased slightly in eyes with DM2 without DR or with non-proliferating DR in more initial stages of the disease (mild/moderate), without reaching statistical significance. Kim et al. [11] and Vujosevic et al. [13] showed that early DR was associated with a thinner choroid compared to the control group.

We analyzed the CT according to the years of evolution of the disease (Group 1 <24 years and Group 2 ≥24 years) with Spectralis SD-OCT and DRI-Triton SS-OCT separately. Significant differences were found in age between the groups, with older age associated with longer evolution of the disease (*p* < 0.001) but with similar ocular characteristics. With the Spectralis SD-OCT, we observed significant differences in three ETDRS areas, I1 and the two vertical perifoveal areas, S2 and I2. With the DRI-Triton SS-OCT, we found differences only in these last two areas (S2 and I2). Spectralis SD-OCT showed vertical areas with greater thicknesses in the less evolved group with differences of 30.36 μm in I1 and 38.92 μm in I2, but the S2 area was thinner by 12.53 μm in the short evolution group which was lower than the differences in the other two areas. The S2 and I2 areas were the only ones with differences using DRI-Triton SS-OCT, with similar results to those obtained with Spectralis OCT: lower thickness values in the long evolution group with a difference in S2 of 27.53 μm less and in I2 of 25.34 μm less compared to the short evolution group. With Spectralis SD-OCT, seven of the nine areas and the CV had lower values in the group with greater evolution. This tendency was maintained with the DRI-Triton SS-OCT in which thinner CTs were obtained in seven of the nine areas in addition to the CV. In summary, CT diminishes with disease evolution as mentioned by other authors [11,12,13,33]. We divided the control group looking for the age influence on our results. We only found differences in the T1 quadrant using Spectralis OCT. We think that part of these differences could be explain by the diminution of choroidal thickness with aging, but this thickening is much clear in patients with DM1.

Malerbi et al. [34] found that the CT was higher in patients with DM1 without DR but with poor metabolic control and an HbA1c higher than 9% compared to controls, and within the group with DM1 the presence of microalbuminuria correlated with increased thickness compared to patients with normal renal function. It is important to assess microalbuminuria as a confounding factor in these patients in addition to those already discussed such as the age and duration of diabetes [13], since both albuminuria and DR have been related to the inflammatory state, endothelial damage or an alteration in the remodelling capacity of extracellular matrix damage [2]. None of our patients had a detectable microalbuminuria and their HbA1c levels were lower than in these studies.

Future longitudinal studies will be required to confirm this result with a larger sample of diabetic patients, without and with DR and with both DM1 and DM2, since their behaviour can differ, influenced by age and other vascular factors. In our population we did not evaluate the choroidal changes with time.

In this study we have only included DM1 without RD, but there are multiple studies showing that the presence of DME can modify CT. Regatieri [23], Abadía [33], Querques [29], Esmaeelpour [12] and Adhi [35] found a reduced choroidal thickness in different areas. Ünsal et al. found that the CT decreases as the disease progresses from a mild to moderate non-proliferative DR, reaching a proliferative DR that correlates to prolonged deficient control of glycaemia and its influence on vascularization [24]. DM is a vascular disease that affects the microcirculation, although it has been less studied for years due to a lack of the necessary instrumentation, and it has to affect the choriocapillaris as well as the layers of medium and large vessels of the choroid. The existence of diabetic choroidopathy has been established [11,23], and CT also diminishes after laser treatment, although modifications in blood flow in the different areas could be found [11,24,36].

Looking for a correlation with the metabolic state, Abadia´s study in DM2 [33] did not detect significant differences according to HbA1c levels or duration of diabetes and CT; there was a moderate correlation between the choroidal thickness and HbA1c levels in patients with DME (r = 0.342, *p* = 0.017). In contrast, Kim et al. [11] found statistically significant differences according to HbA1c levels between the DR groups. They also found a significant correlation between subfoveal CT and HbA1c (r = 0.252, *p* < 0.05). In our study, DM1 patients had a good glycemic control measured by the HbA1c level.

Both Spectralis SD-OCT and DRI-Triton SS-OCT have excellent resolution and are non-invasive devices to evaluate the choroid, with an increased importance in many diseases [19,37]. In DR, they can be very useful to evaluate changes at the choroidal level of blood flow. The relationship between DR and diabetic choroidopathy is not clearly defined in the literature [38]. The choroid supplies oxygen and nutrients to the outer retina. Any change or damage with thinning of this tissue can affect the overlying retina, causing hypoxia and leading to the appearance of retinal lesions or the progression of existing DR. However, it is not known if thinning of the choroid occurs prior to the appearance of DR lesions or if DR lesions are associated with reduced CT. Therefore, knowing the role of the choroid in the retinas of patients with DM and the physiopathological mechanisms involved in DR, including those that affect the choroid, can help us to better understand the course of DR and to optimize the management of the disease based on adapted interventions. Thus, more prospective and longitudinal studies must be carried out.

## 5. Conclusions

In conclusion, the choroid is affected by diabetes. There is a choroidal thinning related to the years of evolution of the disease in DM1 patients without DR. In patients with an evolution of DM greater than 24 years, the CT is statistically lower than in patients with less evolution of the disease.

## Figures and Tables

**Table 1:** Mean, standard deviation (SD) and range (minimum-maximum) of demographics and ocular characteristics of the type 1 diabetes Mellitus (DM1) and control groups. No significant differences (*p* < 0.05) were found between groups in any of the parameters analysed.

	DM1 Group (*n* = 90)	Control Group (*n* = 60)	*p*
Age (years)	41.52 ± 13.05 (22–65)	42.41 ± 13.56 (26–68)	0.361
Refractive error (D)	−1.03 ± 2.23 (−5.00/+ 3.25)	−0.76 ± 2.68 (−5.00/+ 5.00)	0.394
ACD (mm)	3.19 ± 0.51 (2.38–4.10)	3.29 ± 0.33 (2.59–4.00)	0.999
AL (mm)	23.71 ± 2.73 (21.84–26.51)	23.51 ± 1.15 (21.78–26.00)	0.908

ACD, anterior chamber depth; AL, axial length; CT was evaluated in all ETDRS areas and in the total CV in both groups with both OCT systems; Differences were studied in each group using both devices.

**Table 2:** Choroidal thicknesses in the ETDRS quadrants expressed in μm (from the Bruch’s Membrane (BM) to the choroid-scleral limit) measured with the Spectralis spectral domain optical coherence tomography (SD-OCT) and the deep range imaging (DRI)-Triton swept source optical coherence tomography (SS-OCT) in the type 1 diabetes Mellitus (DM1) group and the control group. Values are presented as the mean ± standard deviation and range (minimum-maximum). Significant differences were considered as *p* < 0.05, calculated by Kolmogorov–Smirnov test for two independent nonparametric samples, are shown with *. The volume is represented in mm^3^.

	SPECTRALIS SD-OCT			DRI-TRITON SS-OCT		
	DM1 (*n* = 90)	CONTROL (*n* = 60)	*p*	DM1 (*n* = 90)	CONTROL (*n* = 60)	*p*
Foveal Center (Central ETDRS Region: R1, 1 mm)						
Central R1	352.55 ± 88.20 (126–525)	317.42 ± 80.05 (170–549)	0.004 *	301.30 ± 64.55 (107.17–443.40)	282.32 ± 74.26 (143.01–492.05)	0.025 *
Inner Circle (Parafoveal ETDRS Region: 3 mm)						
Superior S1	351.23 ± 85.20 (160–562)	317.32 ± 71.92 (192–567)	0.003 *	283.18 ± 68.26 (81.35–415.32)	255.81 ± 76.83 (115.78–468.45)	0.014 *
Temporal T1	337.60 ± 80.46 (145–484)	315.59 ± 71.85 (171–536)	0.046 *	272.87 ± 60.20 (106.91–463.28)	263.23 ± 59.71 (141.78–406.01)	0.310
Inferior I1	340.09 ± 88.13 (133–487)	311.12 ± 82.85 (149–557)	0.028 *	225.23 ± 63.27 (71.77–344.04)	202.09 ± 72.93 (84.56–409.92)	0.016 *
Nasal N1	329.70 ± 86.61 (109–501)	292.93 ± 83.58 (145–562)	0.001 *	292.06 ± 61.95 (116.55–463.760)	280.80 ± 66.03 (147.06–466.31)	0.097
Outer Circle (Perifoveal ETDRS Region: 6 mm)						
Superior S2	329.64 ± 81.53 (132–490)	308.72 ± 68.00 (186–552)	0.018 *	297.05 ± 69.55 (105.17–424.30)	278.77 ± 79.90 (129.04–477.19)	0.040 *
Temporal T2	302.07 ± 71.46 (139–469)	287.75 ± 60.22 (182–497)	0.107	288.40 ± 66.21 (110.66–414.31)	273.15 ± 62.76 (155.06–454.61)	0.133
Inferior I2	311.53 ± 87.62 (106–466)	288.95 ± 79.35 (137–561)	0.016 *	272.65 ± 73.86 (91.53–403.04)	263.54 ± 75.29 (109.90–486.64)	0.036 *
Nasal N2	262.60 ± 75.21 (92–410)	232.91 ± 78.20 (115–502)	0.007 *	295.42 ± 64.15 (129.61–433.49)	274.11 ± 62.93 (152.67–457.2)	0.076
Volume	8.82 ± 2.09 (3.62–12.60)	8.12 ± 1.92 (4.48–15)	0.003 *	7.69 ± 1.65 (2.96–11.09)	7.25 ± 1.78 (3.58–12.62)	0.135

SD-OCT, spectral domain optical coherence tomography; DRI, deep range image; SS-OCT, swept source optical coherence tomography; DM, diabetes mellitus; ETDRS, Early Treatment Diabetic Retinopathy Study; R1, central; S, superior; T, temporal; I, inferior; N, nasal. The number 1 corresponds to the 3 mm parafoveal inner circle and 2 to the 6 mm perifoveal outer circle. Significant differences are shown with *.

**Table 3:** Demographics and ocular characteristics of the type 1 diabetes Mellitus (DM1) Group divided depending on the time DM1 evolution: Group 1, less than 24 years and Group 2, more than or equal to 24 years. Values are presented as the mean ± standard deviation and range (minimum–maximum). Significant differences were considered as *p* < 0.05, calculated by Kolmogorov–Smirnov test for two independent nonparametric samples, are shown with *.

	DM1 Group <24 Years (*n* = 46)	DM1 Group ≥24 Years (*n* = 44)	*p*
Age (years)	35.65 ± 12.87 (22–63)	45.59 ± 9.96 (32–65)	<0.001 *
Refractive error (D)	−1.27 ± 2.38 (−5.00/+ 3.25)	−0.78 ± 2.08 (−5.00/+ 3.25)	0.387
ACD (mm)	3.20 ± 0.31 (2.59–3.72)	3.18 ± 0.44 (2.38–4.10)	0.225
AL (mm)	23.63 ± 1.25 (21.84–26.51)	23.78 ± 1.00 (22.24–26.71)	0.070

ACD, anterior chamber depth; AL, axial length.

**Table 4:** Choroidal thicknesses by ETDRS quadrants in μm (from the BM to the choroid-scleral limit) assessed with Spectralis spectral domain optical coherence tomography (SD-OCT) and the deep range imaging (DRI)-Triton swept source optical coherence tomography (SS-OCT) in the type 1 diabetes Mellitus (DM1) group subdivided into two groups: less than 24 years and over or equal to 24 years of evolution of DM1. Values are presented as the mean ± standard deviation and range (minimum–maximum). Significant differences were considered as *p* < 0.05, calculated by Kolmogorov–Smirnov test for two independent nonparametric samples, are shown with *. The volume is represented in mm^3^.

	SPECTRALIS SD-OCT			DRI-TRITON SS-OCT		
	DM <24 (*n* = 46)	DM ≥24 (*n* = 44)	*p*	DM <24 (*n* = 46)	DM ≥24 (*n* = 44)	*p*
**Foveal Center (Central ETDRS Region: R1, 1 mm)**						
Central R1	359,87 ± 83.43 (126–525)	344.91 ± 77.59 (158–485)	0.304	308.67 ± 71.58 (107.17–443.40)	293.59 ± 56.07 (139.10–397.35)	0.152
**Inner Circle (Parafoveal ETDRS Region: 3 mm)**						
Superior S1	346.39 ± 77.73 (160–522)	356.30 ± 77.34 (168–500)	0.783	285.93 ± 75.76 (81.35–415.32)	280.31 ± 60.17 (123.97–374.02)	0.692
Temporal T1	347.47 ± 73.55 (145–484)	327.27 ± 71.09 (156–481)	0.238	280.07 ± 61.93 (115.20–463.28)	265.34 ± 58.08 (106.91–342.31)	0.434
Inferior I1	354.93 ± 82.00 (136–487)	324.57 ± 77.96 (133–485)	0.031 *	224.40 ± 66.39 (73.44–344.04)	226.10 ± 60.59 (71.77–326.64)	0.881
Nasal N1	333.54 ± 85.37 (109–507)	325.68 ± 74.44 (137–452)	0.557	299.75 ± 68.46 (116.55–463.76)	284.04 ± 53.95 (125.83–367.94)	0.178
**Outer Circle (Perifoveal ETDRS Region: 6 mm)**						
Superior S2	323.52 ± 77.85 (158–490)	336.05 ± 70.62 (132–465)	0.044 *	310.51 ± 71.24 (105.17–424.30)	282.98 ± 65.60 (121.24–398.88)	0.016 *
Temporal T2	313.13 ± 64.14 (139–469)	290.52 ± 63.38 (140–433)	0.166	279.68 ± 68.15 (132.03–414.31)	297.51 ± 63.62 (110.66–412.02)	0.063
Inferior I2	330.56 ± 82.35 (126–466)	291.64 ± 77.12 (106–436)	0.036 *	285.04 ± 72.33 (91.53–371.42)	259.70 ± 74.04 (93.75–403.40)	0.045 *
Nasal N2	263.61 ± 72.50 (100–378)	261.55 ± 68.92 (92–410)	0.981	296.36 ± 70.69 (129.61–433.49)	294.43 ± 57.31 (145.27–389.12)	0.977
Volume	9.03 ± 81.95 (3.89–12.58)	8.64 ± 1.78 (3.62–12.60)	0.708	7.78 ± 1.74 (3.02–11.09)	7.58 ± 1.56 (2.96–9.94)	0.523

SD-OCT, spectral domain optical coherence tomography; DRI, deep range image; SS-OCT, swept source optical coherence tomography; DM, diabetes mellitus; ETDRS, Early Treatment Diabetic Retinopathy Study; R1, central; S, superior; T, temporal; I, inferior; N, nasal. The number 1 corresponds to the 3 mm parafoveal inner circle and 2 to the 6 mm perifoveal outer circle. Significant differences are shown with *.

## References

[1] Kempen J.H., O’Colmain B.J., Leske M.C., Haffner S.M., Klein R., Moss S.E., Taylor H.R., Hamman R.F., Group EDPR (2004). The prevalence of diabetic retinopathy among adults in the United States. Arch Ophthalmol..

[2] Nickla D.L., Wallman J. (2010). The multifunctional choroid. Prog Retin Eye Res..

[3] Spaide R.F., Koizumi H., Pozzoni M.C., Pozonni M.C. (2008). Enhanced depth imaging spectral-domain optical coherence tomography. Am. J. Ophthalmol..

[4] Coscas G., Zhou Q., Coscas F., Zucchiatti I., Rispoli M., Uzzan J., De Benedetto U., Savastano M.C., Soules K., Goldenberg D. (2012). Choroid thickness measurement with RTVue optical coherence tomography in emmetropic eyes, mildly myopic eyes, and highly myopic eyes. Eur. J. Ophthalmol..

[5] Michalewska Z., Michalewski J., Nawrocki J. (2013). New OCT technologies take imaging deeper and wider. Adding the possibility of imaging the choroid, retina, and vitreous. Retin. Physician.

[6] Maruko I., Iida T., Sugano Y., Oyamada H., Sekiryu T., Fujiwara T., Spaide R.F. (2011). Subfoveal choroidal thickness after treatment of Vogt-Koyanagi-Harada disease. Retina.

[7] Maruko I., Iida T., Sugano Y., Ojima A., Sekiryu T. (2011). Subfoveal choroidal thickness in fellow eyes of patients with central serous chorioretinopathy. Retina.

[8] Maruko I., Iida T., Sugano Y., Oyamada H., Akiba M., Sekiryu T. (2012). Morphologic analysis in pathologic myopia using high-penetration optical coherence tomography. Invest. Ophthalmol. Vis. Sci..

[9] Shao L., Xu L., Chen C.X., Yang L.H., Du K.F., Wang S., Zhou J.Q., Wang Y.X., You Q.S., Jonas J.B. (2013). Reproducibility of subfoveal choroidal thickness measurements with enhanced depth imaging by spectral-domain optical coherence tomography. Invest. Ophthalmol. Vis. Sci..

[10] Melancia D., Vicente A., Cunha J.P., Abegão Pinto L., Ferreira J. (2016). Diabetic choroidopathy: A review of the current literature. Graefes Arch. Clin. Exp. Ophthalmol..

[11] Kim J.T., Lee D.H., Joe S.G., Kim J.G., Yoon Y.H. (2013). Changes in choroidal thickness in relation to the severity of retinopathy and macular edema in type 2 diabetic patients. Invest. Ophthalmol. Vis. Sci..

[12] Esmaeelpour M., Považay B., Hermann B., Hofer B., Kajic V., Hale S.L., North R.V., Drexler W., Sheen N.J. (2011). Mapping choroidal and retinal thickness variation in type 2 diabetes using three-dimensional 1060-nm optical coherence tomography. Invest. Ophthalmol. Vis. Sci..

[13] Vujosevic S., Martini F., Cavarzeran F., Pilotto E., Midena E. (2012). Macular and peripapillary choroidal thickness in diabetic patients. Retina.

[14] Cunha-Vaz J., Faria de Abreu J.R., Campos A.J. (1975). Early breakdown of the blood-retinal barrier in diabetes. Br. J. Ophthalmol..

[15] Ciulla T.A., Harris A., Latkany P., Piper H.C., Arend O., Garzozi H., Martin B. (2002). Ocular perfusion abnormalities in diabetes. Acta Ophthalmol. Scand..

[16] Early Treatment Diabetic Retinopathy Study Research G (1985). Photocoagulation for diabetic macular edema. Arch. Ophthalmol..

[17] Orduna E., Sanchez-Cano A., Luesma M.J., Perez-Navarro I., Abecia E., Pinilla I. (2018). Interocular symmetry of choroidal thickness and volume in healthy eyes on optical coherence tomography. Ophthalmic. Res..

[18] Sanchez-Cano A., Orduna E., Segura F., Lopez C., Cuenca N., Abecia E., Pinilla I. (2014). Choroidal thickness and volume in healthy young white adults and the relationships between them and axial length, ammetropy and sex. Am. J. Ophthalmol..

[19] Agawa T., Miura M., Ikuno Y., Makita S., Fabritius T., Iwasaki T., Goto H., Nishida K., Yasuno Y. (2011). Choroidal thickness measurement in healthy Japanese subjects by three-dimensional high-penetration optical coherence tomography. Graefes Arch. Clin. Exp. Ophthalmol..

[20] Barteselli G., Chhablani J., El-Emam S., Wang H., Chuang J., Kozak I., Cheng L., Bartsch D.U., Freeman W.R. (2012). Choroidal volume variations with age, axial length, and sex in healthy subjects: A three-dimensional analysis. Ophthalmology.

[21] Herrera L., Perez-Navarro I., Sanchez-Cano A., Perez-Garcia D., Remon L., Almenara C., Caramello C., Cristóbal J.A., Pinilla I. (2015). Choroidal thickness and volume in a healthy pediatric population and its relationship with age, axial length, ametropia, and sex. Retina.

[22] Li X.Q., Larsen M., Munch I.C. (2011). Subfoveal choroidal thickness in relation to sex and axial length in 93 Danish university students. Invest. Ophthalmol. Vis. Sci..

[23] Regatieri C.V., Branchini L., Carmody J., Fujimoto J.G., Duker J.S. (2012). Choroidal thickness in patients with diabetic retinopathy analyzed by spectral-domain optical coherence tomography. Retina.

[24] Unsal E., Eltutar K., Zirtiloğlu S., Dinçer N., Ozdoğan Erkul S., Güngel H. (2014). Choroidal thickness in patients with diabetic retinopathy. Clin. Ophthalmol..

[25] Philip A.M., Gerendas B.S., Zhang L., Faatz H., Podkowinski D., Bogunovic H., Abramoff M.D., Hagmann M., Leitner R., Simader C. (2016). Choroidal thickness maps from spectral domain and swept source optical coherence tomography: Algorithmic versus ground truth annotation. Br. J. Ophthalmol..

[26] Tavares Ferreira J., Proença R., Alves M., Dias-Santos A., Santos B.O., Cunha J.P., Papoila A.L., Abegão Pinto L. (2017). Retina and choroid of diabetic patients without observed retinal vascular changes: A longitudinal study. Am. J. Ophthalmol..

[27] Esmaeelpour M., Brunner S., Ansari-Shahrezaei S., Shahrezaei S.A., Nemetz S., Povazay B., Kajic V., Drexler W., Binder S. (2012). Choroidal thinning in diabetes type 1 detected by 3-dimensional 1060 nm optical coherence tomography. Invest. Ophthalmol. Vis. Sci..

[28] Esmaeelpour M., Povazay B., Hermann B., Hofer B., Kajic V., Kapoor K., Sheen N.J., North R.V., Drexler W. (2010). Three-dimensional 1060-nm OCT: Choroidal thickness maps in normal subjects and improved posterior segment visualization in cataract patients. Invest. Ophthalmol. Vis. Sci..

[29] Querques G., Lattanzio R., Querques L., Del Turco C., Forte R., Pierro L., Souied E.H., Bandello F. (2012). Enhanced depth imaging optical coherence tomography in type 2 diabetes. Invest. Ophthalmol. Vis. Sci..

[30] Xu J., Xu L., Du K.F., Shao L., Chen C.X., Zhou J.Q., Wang Y.X., You Q.S., Jonas J.B., Wei W.B. (2013). Subfoveal choroidal thickness in diabetes and diabetic retinopathy. Ophthalmology.

[31] Muir E.R., Rentería R.C., Duong T.Q. (2012). Reduced ocular blood flow as an early indicator of diabetic retinopathy in a mouse model of diabetes. Invest. Ophthalmol. Vis. Sci..

[32] Nagaoka T., Kitaya N., Sugawara R., Yokota H., Mori F., Hikichi T., Fujio N., Yoshida A. (2004). Alteration of choroidal circulation in the foveal region in patients with type 2 diabetes. Br. J. Ophthalmol..

[33] Abadia B., Suñen I., Calvo P., Bartol F., Verdes G., Ferreras A. (2018). Choroidal thickness measured using swept-source optical coherence tomography is reduced in patients with type 2 diabetes. PLoS ONE.

[34] Malerbi F.K., Regatieri C.V., de Sa J.R., Morales P.H., Farah M.E., Dib S.A. (2018). Microalbuminuria is associated with increased choroidal thickness in type 1 diabetes mellitus patients without diabetic retinopathy. Acta Ophthalmol..

[35] Adhi M., Brewer E., Waheed N.K., Duker J.S. (2013). Analysis of morphological features and vascular layers of choroid in diabetic retinopathy using spectral-domain optical coherence tomography. JAMA Ophthalmol..

[36] Cho G.E., Cho H.Y., Kim Y.T. (2013). Change in subfoveal choroidal thickness after argon laser panretinal photocoagulation. Int. J. Ophthalmol..

[37] Margolis R., Spaide R.F. (2009). A pilot study of enhanced depth imaging optical coherence tomography of the choroid in normal eyes. Am. J. Ophthalmol..

[38] Rayess N., Rahimy E., Ying G.S., Bagheri N., Ho A.C., Regillo C.D., Vander J.F., Hsu J. (2015). Baseline choroidal thickness as a predictor for response to anti-vascular endothelial growth factor therapy in diabetic macular edema. Am. J. Ophthalmol..

